# Uncommon unilateral congenital knee dislocation. A case report

**DOI:** 10.1016/j.ijscr.2025.110958

**Published:** 2025-01-25

**Authors:** Adugnaw Bogale Worku, Molla Asnake Kebede, Hashime Meketa Negatie, Alemayehu Beharu Tekle, Alemayehu Dagne Abate, Solyana Haileselassie Admassie

**Affiliations:** a Department of Orthopedics Surgery, School of Medicine, College of Medicine and Health Sciences, Mizan-Tepi University, Mizan-Teferi, Ethiopia; b Department of Medicine, School of Medicine, College of Medicine and Health Sciences, Mizan-Tepi University, Mizan-Teferi, Ethiopia; c Department of Radiology, School of Medicine, College of Medicine and Health Sciences, Mizan-Tepi University, Mizan-Teferi, Ethiopia; d Department of Emergency and Critical care medicine, School of Medicine, College of Medicine and Health Sciences, Mizan-Tepi University, Mizan-Teferi, Ethiopia; e Department of Radiology, Eftu General Hospital, Dire Dawa, Ethiopia; f Department of Radiology, Mehal Meda Hospital, Mehal Meda, Ethiopia

**Keywords:** Dislocation, Congenital, Knee, Neonatal, Genu recurvatum

## Abstract

**Introduction and importance:**

The estimated incidence of congenital dislocation of the knee, also referred to as genu recurvatum, is approximately 1 in 100,000 live births. The purpose of this report is to present a rare case of unilateral congenital knee dislocation, highlighting the clinical presentation and management.

**Case presentation:**

A 9-day-old female infant was born to a 30-year-old primigravida mother following an uncomplicated term pregnancy of 39 weeks and 4 days. Upon examination at the hospital, the infant presented with a hyperextended left knee. Additionally, there was a deep suprapatellar skin crease. A conservative treatment plan was initiated, involving serial long-leg casting and positioning of the foot to establish a plantigrade position.

**Discussion:**

Congenital knee dislocation presents at birth with hyperextension and can limit knee movement. Diagnosis involves clinical examination and imaging to assess severity. Treatment varies by grade, with conservative methods like casting for mild cases and surgery for severe ones. Early intervention is critical for better outcomes. In this case, a Grade I congenital dislocation of the knee was successfully treated with reduction and casting.

**Conclusion:**

In conclusion, this case report stands as a valuable addition to the medical literature, offering insights into a rare presentation of unilateral congenital knee dislocation and emphasizing the importance of early diagnosis and intervention in similar cases.

## Highlights

• Early identifying CDK in newborns is important for better treatment outcomes.
• Conservative management using serial long-leg casting to improve knee alignment
• Regular monitoring is crucial to ensure correction and prevent complications.
• Favorable outcomes are achievable with appropriate care.

## 1 Introduction

The estimated incidence of congenital dislocation of the knee (CDK), also known as genu recurvatum, is approximately 1 in 100,000 live births, making it a rare condition. Less frequent than other congenital musculoskeletal disorders like developmental dysplasia of the hip. It is more common in females than males, and familial patterns suggest a possible genetic component, though specific genetic factors remain unclear. Understanding its rarity and associations is important for early diagnosis and management to prevent long-term functional impairment [1,2].

.The pathogenesis and hereditary patterns of CKD are not fully understood, with most cases being familial. CKD is usually managed immediately after birth [3]. CDK is often associated with other musculoskeletal anomalies, among which DDH is the most common deformity [4,5]. CND is recognized either on prenatal ultrasound or immediately after birth and is characterized by hyperextension deformity at the knee [6].

The existing literature on CDK is sparse. Consequently, there is a lack of comprehensive guidance on optimal treatment approaches, particularly for non-syndromic cases. This knowledge gap emphasizes the necessity for further reports to inform clinical decision-making. This report seeks to reduce this gap by presenting a detailed case study of CDK, underscoring the importance of early diagnosis and timely intervention in achieving favorable outcomes. Furthermore, it aims to assist clinicians in enhancing their understanding and management of this rare condition.

## 2 Clinical presentation

A 9-day-old female infant was born to a 30-year-old primigravida mother following an uncomplicated term pregnancy of 39 weeks and 4 days. Antenatal care adhered to standard guidelines, with no abnormalities detected on ultrasound. Both mother and infant had favorable outcomes after a normal spontaneous vaginal delivery at a local health facility. The newborn had APGAR scores of 7 and 8 and weighed 2.8 kg. However, an abnormal extension of the left knee was noted, prompting a referral to our hospital's orthopedic clinic 9 days after delivery.

**Fig. 1:**
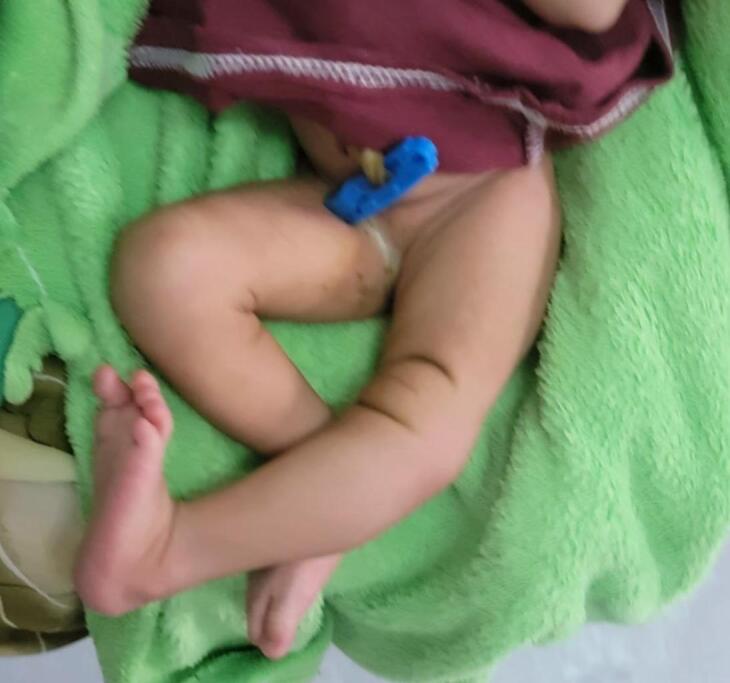
Hyperextended left knee joint.

**Fig. 2:**
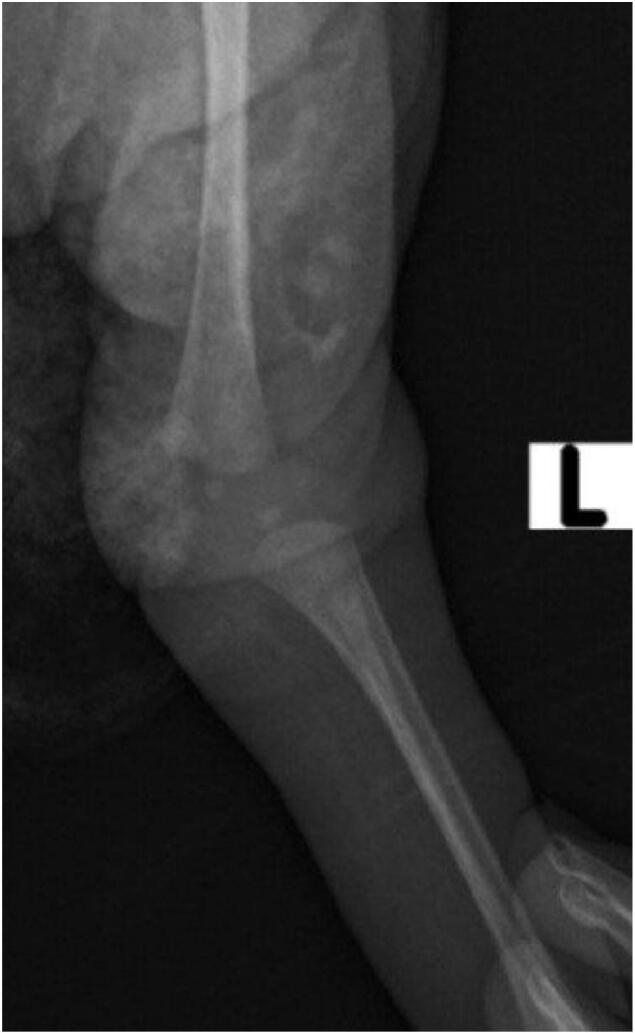
Initial left knee AP X-ray showing left knee dislocation.

Upon examination at the hospital, the infant presented with hyperextended left knee, positioned at a 30-degree angle (Fig. 1). Additionally, there was a deep suprapatellar skin crease. Passive flexion of the knee was limited to about 10 degrees. Despite this, examinations of the spine, hips, and feet revealed no abnormalities. Initial X-ray of left knee Showed left knee dislocation (Fig. 2).

**Fig. 3:**
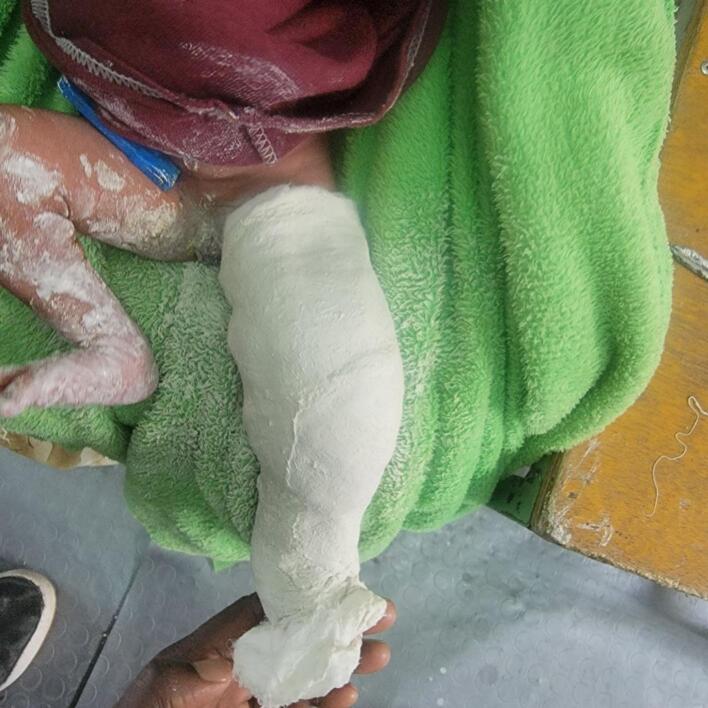
Knee position after first long leg casting.

**Fig. 4:**
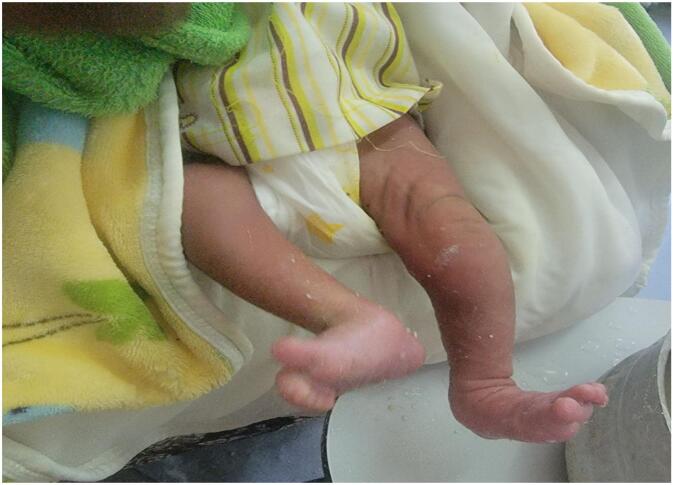
The left knee position after one week of casting.

**Fig. 5:**
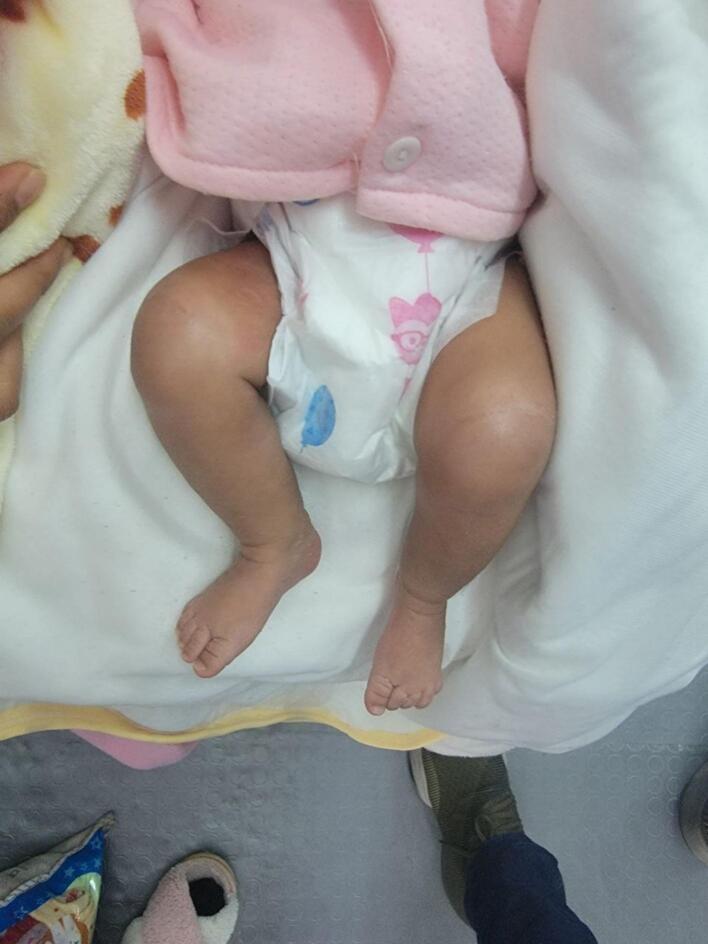
Normal appearing knee after third long leg cast.

A conservative treatment plan was initiated, involving serial long-leg casting and positioning of the foot to establish a plantigrade position. A reduction maneuver was performed, followed by serial casting. This procedure involved flexing the hip to 90 degrees while carefully manipulating the foot and thigh to realign the knee. The knee was brought to a neutral position (0 degrees of flexion/extension) and then gradually flexed. Over the course of treatment, the infant underwent serial long-leg casting, achieving 30 degrees of flexion in the first week (Fig. 3, Fig. 4), 50 degrees in the second week and 90 degrees in the third week. After cast removal, the left knee maintained a 90-degree flexed position, with passive flexion comparable to the opposite knee (Fig. 5).

**Fig. 6:**
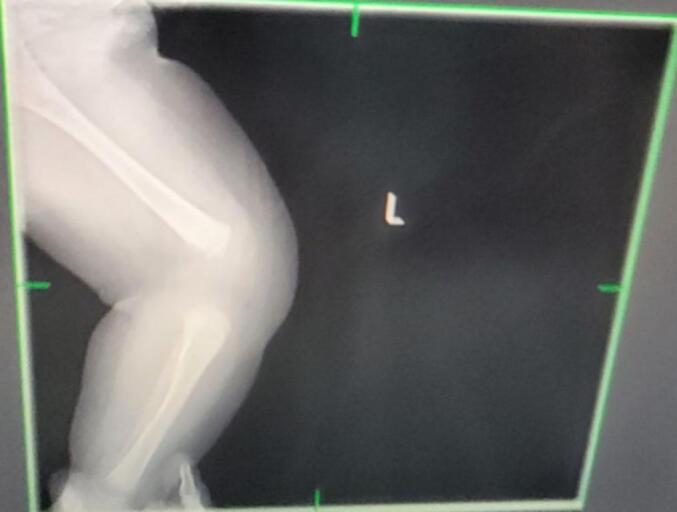
X-ray of left knee showing normal finding.

X-ray of left knee after third casting showed normal finding (Fig. 6).

The treatment lasted three weeks, and no further interventions, such as bracing, physiotherapy, or additional manipulation, were required. Follow-up evaluations at 9 and 12 weeks confirmed successful correction of the knee deformity, with normal range of motion, alignment, and stability restored to the affected knee joint.

## 3 Discussion

Congenital knee dislocation was first described by Chanssier in 1812 [7]. The reported incidence is approximately 0.017 per 1000 live births, with no significant difference between the right and left knees [5,6]. This uncommon condition is often associated with various skeletal abnormalities and syndromic complexes, although its exact cause remains unclear. Three main theories have been proposed to explain this rare condition. Mechanical factors include fetal malposition, such as leg entrapment under the mandible, oligohydramnios, prolonged breech position, primary quadriceps tendon contracture due to fibrosis, and anterior cruciate ligament malformation [8,9]. Intrinsic or genetic factors are also implicated, such as in Larsen syndrome, which occurs in about 1 per 100,000 live births and is linked to mutations in the filamin B gene [2,5]. Additionally, congenital knee dislocation may be associated with other musculoskeletal abnormalities, such as congenital hip dysplasia. In our case, the fetal presentation was cephalic, the amniotic fluid index was normal, and there were no visible signs suggesting an associated tendon or ligament disorder. MRI for detailed evaluation of tendon and ligament malformations, as well as genetic testing, were not performed due to the unavailability of these tests in our facility and the patient's inability to afford referral to a higher center. However, the infant had no associated dysplasia or syndrome, such as Larsen syndrome or hip dysplasia. The only possible risk factor we identified is being female.

Congenital knee dislocation (CDK) commonly presents at birth with visible hyperextension of the knee, where the joint may appear bent backward. Severe cases may show limited or absent knee flexion due to dislocation. If untreated, CDK can lead to abnormal gait patterns and compensatory movements as the child grows. The condition is often associated with other congenital anomalies, such as hip dysplasia and Larsen syndrome. Clinically, the knee may feel unstable, and muscle imbalances, particularly quadriceps contractures, can further restrict movement [10,11]. In our case the infant was presented hyperextended left knee joint.

In 2011, Abdelaziz and Samir developed the CDK grading system, emphasizing passive knee flexion as a key indicator of severity and treatment guidance. GI is defined by over 90 degrees of passive flexion, GII by 30–90 degrees, and GIII by less than 30 degrees [12,13]. In our case, we used Abdelaziz and Semir classification and it was classified as Grade I, as the infant initially presented with a hyperextended left knee positioned at 30 degrees and passive flexion limited to 10 degrees. There was progressive improvement with serial casting, bringing the knee to a neutral position and eventually achieving 90 degrees of flexion.

The diagnosis of CKD primarily involves a thorough clinical examination and imaging studies. At birth, the condition is often identified through the observation of hyperextension or a bent appearance of the knee. Physical examination may reveal limited or absent knee flexion, instability, and potential muscle imbalances. Imaging techniques, such as X-rays, play a supportive role in assessing the alignment of the tibia and femur, determining the degree of dislocation, and ruling out any associated skeletal abnormalities. In some cases, ultrasound may also be utilized to evaluate soft tissue structures and further assess the knee joint. Early diagnosis is essential for effective management and to prevent complications associated with untreated CKD [10,14,15]. In our case, the diagnosis was made clinically, with both the tibia and femur aligned, and no associated skeletal abnormalities present. Additionally, the ultrasound findings were unremarkable.

Management of congenital knee dislocation using the Abdulaziz classification is tailored based on the severity of the dislocation. For Grade I, the approach is conservative. This typically includes initiating serial casting or splinting to maintain proper alignment and support the knee joint, along with encouraging gentle range-of-motion exercises to promote flexibility and strength. Regular follow-up is essential to monitor progress. In Grade II, the management involves applying serial long-leg casting to gradually improve alignment and flexion. Reduction maneuvers may also be performed to realign the knee before casting, and physical therapy is incorporated to enhance mobility. For Grade III, surgical intervention is often required due to the irreducibility of the dislocated knee. This may involve tendon release, capsular repair, or realignment procedures. Postoperatively, the knee may be immobilized, followed by rehabilitation to restore function. Regardless of the grade, early diagnosis and intervention are crucial for optimal outcomes, and regular follow-ups are essential to monitor progress and make necessary adjustments to the treatment plan [12,13,17]. In our case, the patient presented early with Grade I congenital knee dislocation; therefore, a reduction maneuver was performed, followed by serial casting. This procedure involved flexing the hip to 90 degrees while carefully manipulating the foot and thigh to realign the knee. The outcome was successful, with significant improvement in knee alignment and function. The work has been reported in line with the SCARE criteria [18].

## 4 Conclusion

In conclusion, this case report stands as a valuable addition to the medical literature, offering insights into a rare presentation of unilateral congenital knee dislocation and emphasizing the importance of early diagnosis and intervention in similar cases.

## CRediT authorship contribution statement

Adugnaw Bogale Worku, MD: Play major role in patient management. Involved in the conception and design of the study, drafting and revising of the article and final approval of the version to be submitted and also involved in direct management of the patient.
Molla Asnake Kebede, MD: Involved in the conception and design of the study, drafting and revising of the article and final approval of the version to be submitted and also involved in direct management of the patient.
Hashime Meketa Negatie: Involved in the conception and design of the study, drafting and revising of the article and final approval of the version to be submitted and also involved in direct management of the patient.
Alemayehu Beharu Tekle, MD: Involved in the conception and design of the study, drafting and revising of the article and final approval of the version to be submitted and also involved in direct management of the patient.
Alemayehu Dagne Abate: Involved in the conception and design of the study, drafting and revising of the article and final approval of the version to be submitted and also involved in direct management of the patient.
Solyana Haileselassie Admassie: Involved in the conception and design of the study, drafting and revising of the article and final approval of the version to be submitted and also involved in direct management of the patient.

## Informed consent

Written informed consent was obtained from the patient's parents for publication and any accompanying images. A copy of the written consent is available for review by the Editor-in-Chief of this journal on request.

## Ethical approval

Ethical approval for this study was obtained from the College Research Committee and Reference No. HSE/00429/2016 on 12 September 2024.

## Guarantor

Molla Asnake Kebede.

## Research registration number

Not applicable.

## Funding

The case report, authorship, and/or publication of this work were done without outside funding.

## Declaration of competing interest

All authors declared no conflicts of interest in this work.

## Data Availability

On a valid request, the corresponding author will provide access to the datasets that were gathered and used to conduct this article.
